# Co-Transplantation of Olfactory Ensheathing Cells from Mucosa and Bulb Origin Enhances Functional Recovery after Peripheral Nerve Lesion

**DOI:** 10.1371/journal.pone.0022816

**Published:** 2011-08-03

**Authors:** Nicolas Guérout, Alexandre Paviot, Nicolas Bon-Mardion, Célia Duclos, Damien Genty, Laetitia Jean, Olivier Boyer, Jean-Paul Marie

**Affiliations:** 1 Experimental Surgery Laboratory, Groupe de Recherche sur le Handicap Ventilatoire (GRHV), UPRES EA 3830, Institut de Recherche et d'Innovation Biomédicale de Haute Normandie (IRIB), Faculty of Medicine and Pharmacy, University of Rouen, Rouen, France; 2 Otorhinolaryngology, Head and Neck Surgery Department, Rouen University Hospital, Charles Nicolle Hospital, Rouen, France; 3 Pathology Laboratory-Pavillon Jacques Delarue, Rouen University Hospital, Charles Nicolle Hospital, Rouen, France; 4 INSERM U905, Faculty of Medicine and Pharmacy, Institute for Medical Research, University of Rouen, Rouen, France; Universidade Federal do Rio de Janeiro, Brazil

## Abstract

Olfactory ensheathing cells (OECs) represent an interesting candidate for cell therapy and could be obtained from olfactory mucosa (OM-OECs) or olfactory bulbs (OB-OECs). Recent reports suggest that, depending on their origin, OECs display different functional properties. We show here the complementary and additive effects of co-transplanting OM-OECs and OB-OECs after lesion of a peripheral nerve. For this, a selective motor denervation of the laryngeal muscles was performed by a section/anastomosis of the recurrent laryngeal nerve (RLN). Two months after surgery, recovery of the laryngeal movements and synkinesis phenonema were analyzed by videolaryngoscopy. To complete these assessments, measure of latency and potential duration were determined by electrophysiological recordings and myelinated nerve fiber profiles were defined based on toluidine blue staining. To explain some of the mechanisms involved, tracking of GFP positive OECs was performed. It appears that transplantation of OM-OECs or OB-OECs displayed opposite abilities to improve functional recovery. Indeed, OM-OECs increased recuperation of laryngeal muscles activities without appropriate functional recovery. In contrast, OB-OECs induced some functional recovery by enhancing axonal regrowth. Importantly, co-transplantation of OM-OECs and OB-OECs supported a major functional recovery, with reduction of synkinesis phenomena. This study is the first which clearly demonstrates the complementary and additive properties of OECs obtained from olfactory mucosa and olfactory bulb to improve functional recovery after transplantation in a nerve lesion model.

## Introduction

Olfactory ensheathing cells (OECs) are specialized glial cells that guide the regeneration of non-myelinated olfactory axons from the peripheral nasal epithelium through the cribriform plate of the ethmoid bone and into the olfactory bulb (OB) [1], [2]. Several studies have demonstrated the great potential of OECs to improve functional recovery and axonal regrowth after lesions of the CNS or PNS [3], [4], [5]. The source of OECs dramatically influences the success of cellular transplantation. In fact, it seems that OECs from OB and OM are specialized in different physiological processes [6], [7], [8], and have a different behavior after transplantation *in vivo* [7]. Recently, we demonstrated in a vagus nerve (VN) lesion model, that both type of OECs could improve electrical muscular activity and nervous conduction, but that OM-OECs induce aberrant movements, whereas OB-OECs increase functional recovery [9]. It appears too, that OM-OECs enhance tissue healing and reduce fibrosis. These two specific abilities could be crucial to improve recovery after lesion of the nervous system.

We report herein additive effects of co-injected OECs from OB and OM on selective reinnervation of laryngeal muscles after a section/anastomosis of the recurrent laryngeal nerve.

## Materials and Methods

### Animals

Experiments were performed at the Laboratory of Experimental Surgery, Faculty of Medicine, Rouen, France (University license: B76-450-05, surgeon license: 76.A.21), after acceptance by the Ethical Committee for Animal Experiments in Normandy: N/01-01- 10/01/01-13. All surgery was performed under ketamine hydrochloride and chlorpromazine hydrochloride anesthesia, all efforts were made to minimize suffering.

For this study Fischer inbred rats were used (Charles River, L'Arbresle, France).

Individual rats were identified by implantation of GLASS TAG (Reseaumatique, Bernay, France).

### Cell culture

Olfactory bulb and olfactory mucosa primary cultures were prepared as described previously by our team [8]. Briefly, rats were deeply anesthetized (isofluorane) and decapitated. OB were immediately dissected and placed in Hank's buffered salt solution (H.B.S.S, Invitrogen, Carlsbad, CA), after removing the meninges. H.B.S.S containing 0.1% trypsin (Invitrogen) and OB were incubated for 45 min at 37°C. Trypsinization was stopped by adding Dulbecco's Modified Eagle's/Ham's F12 medium (D.M.E.M/F12, Invitrogen), supplemented with 10% Fetal Bovine Serum (F.B.S, Invitrogen) and 1% penicillin/streptomycin (Invitrogen) (DF-10S). The tissue was centrifuged at 1.000 RPM for 3 min, resuspended in DF-10S and centrifuged again. Tissue was then triturated in 2 ml DF-10S using a micropipette, until an homogenous cell suspension was obtained. Cells were plated in DF-10S in 75 cm^2^ flasks (TPP, Trasadingen, Switzerland), pre-coated with poly-L-Lysine (50 µg/mL). The flasks were incubated at 37°C, 5% CO_2_. The medium was changed every two days. Eight days after plating, OB primary cultures were confluent.

In the same rats, the nasal cavity was opened sagittally with a spatula and OM was cut into small pieces. After a short rinse in H.B.S.S, samples were trypsinized (1% trypsin for 45 min at 37°C) (Invitrogen). The OM is easily identified in the rat by its posterior position on the nasal septum and by the yellowish appearance of the epithelial surface.

Then, samples were cultivated with the same protocol that for OB cultures [10].

Cultures were checked for OECs by immunocytochemistry and flow-cytometry, as previously described [8]. These analyses permitted to determinate that after eight days *in vitro* OB cultures contained approximately 50% of p75 positive cells, whereas in the same time OM cultures contained about 10% of p75 positive cells.

#### Preparation of the cells for grafting

Before transplantation, cultures were trypsinized to remove them from the dishes, and the cells were counted using a hemocytometer. OB and OM cultures cells were resuspended in a 30∶60 (v/v) solution of DMEM/F-12∶Matrigel (BD Biosciences, San Jose, CA.) immediately before grafting. Each animal received a total of 6×10^5^ OECs for transplantation.

### Experimental nerve lesion and cell transplantation procedure

For experimental groups, adult male Fischer inbred rats (225–250 g) were used.

All the rats were anesthetized by intraperitoneal injection of ketamine hydrochloride (12.5 mg/kg) and chlorpromazine hydrochloride (0.625 mg/kg).

Five groups were classified as follows:

Group 1 (n = 10): sham control group, the right recurrent laryngeal nerve (RLN) was only surgically exposed, but not injured.

Group 2 (n = 10): the RLN was sectioned and sutured without treatment (reinnervated control group).

For the groups 3 (n = 10), 4 (n = 10) and 5 (n = 10), the right RLN was exposed and cut at the level of the seventh tracheal ring. Then, anastomosis was done by one point of 11.0 wire (Ethicon, Somerville, NJ) under microscopic control (Zeiss, Oberkochen, Germany). This surgery constituted a selective motor denervation of the laryngeal muscles, RLN is a mixed nerve.

For these groups, using a micropipette, 90 µl DMEM/F-12∶Matrigel (30∶60) containing 6×10^6^ cells (6×10^5^ OECs) of OM origin (group 3: reinnervated-OM treated group) or 1.2×10^6^ cells (6×10^5^ OECs) of OB origin (group 4: reinnervated-OB treated group), or a mixture of 6×10^5^ cells of OB origin (3×10^5^ OECs) and 3×10^6^ cells of OM origin (3×10^5^ OECs) (group 5: reinnervated-OM+OB treated group) cultures was laid over the section/anastomosis site, immediately at the time of surgery. The same numbers of OECs was injected in all the treated groups.

Matrigel was used for cellular transplantation because when incubated at 37°C (body temperature), the Matrigel proteins self-assemble producing a thin film which permits to retain OECs on site of injection. Care was taken that Matrigel containing OECs was self-assembled on anastomosed nerve before muscle and skin were closed. To this manner, 2 cm of RLN were recovered by Matrigel.

After surgery, all the rats were caged individually under a heat lamp during 24 hours.

Before and after the experiments, rats were kept on standard laboratory food and tap water ad libitum, with an artificial 12 hours light/dark cycle.

### Evaluation

Evaluations were performed 2 months after surgery, under spontaneous ventilation, on 10 rats of each group. All the investigations were performed blindly.

#### Videolaryngoscopy

The videolaryngoscopic evaluation was performed as previously described, in using a video camera (Telecam SL NLSC 20212120, Karl Storz Endoskope) [9].

The 30° laryngoscope (Karl Storz GmbH & Co. KG, Tuttlingen, Germany) was inserted orally and adjusted to provide the best view of the larynx. Anatomic landmarks were determined to reproduce the same view for each recording [11].

Three sequences of two glottal frames were selected for each rat, consisting in the images of successive maximal adduction and maximal abduction. For each sequence absolute angular differences was measured between movement of maximal abduction (Figure S1A) and maximal adduction (Figure S1B) for the right vocal cord.

To complete the analysis of the angular difference by dynamic measure, the vocal cords movement during inspiration was scored. Dynamic score was based on movement amplitude. In terms of amplitude, for each recording, we scored 0 for no movement, 1 for minimal amplitude of movement, 2 for important amplitude of movement with a total adduction and 3 for normal amplitude of movement.

To complete this measurement, in order to score the functional recovery of larynx, synkinesis and paradoxal movements were observed. For this, in using another scale in which all paradoxal movements were scored with 0, functional recovery was performed. In terms of function, for each recording, we scored 0 for no or paradoxal movement, 1 for a minimal functional movement, 2 for an important functional movement and 3 for a total recuperation of laryngeal function [12].

For these purposes, comparison to the healthy side served as reference.

#### Electromyography (EMG)

The electrophysiological evaluafotion was performed as previously described [9]. Anesthetized rat was placed in a dorsal decubitus position, and a median incision was made to expose the larynx. To record the right posterior cricoarytenoid muscle (PCA) activity, a monopolar needle electrode (38×0.45 mm) (AMBU, Gouda, Netherlands) was introduced in the PCA, after an incision of the cricothyroid membrane. Samplings were displayed on an EMG recorder (Electromyogram Keypoint, Dantec Dynamics, Bristol, UK).

The traces were recorded and the muscular activity of PCA was analyzed in terms of richness and synchronization with respiration.

Then, the right VN was dissected and stimulated during 0.1 ms with a progressive increase of the intensity. The electrical response in the PCA muscle was recorded and latency and potential duration were measured for the supramaximal amplitude of stimulation.

VN was chosen for site of stimulation, because the size of the RLN could not permit to position the stimulator without inducing nerve lesion.

#### Morphometric analysis for axonal counting

For this evaluation, rats were euthanized (overdose of pentobarbital), then, RLN was sectioned and removed.

The distal portions of the RLN were fixed, blocked, and embedded as previously described by Roglio et al. [13].

Then, semi-thin transverse sections (1 μm-thick) were cut using a Pyramitome Ultramicrotomy System (Ultracut S, Reichert/Leica, Solms, Germany) and stained by 0.1% Toluidine Blue in 1% sodium tetraborate (Merck, Darmstadt, Germany) for 75 s at 70°C.

The total number of fibers was counted in the RLN (magnification: ×630) by use of an image analysis system (Mercator, Explora Nova, La Rochelle, France).

In each RLN, myelinated nerve fiber profiles were counted and measured by determining the outer and inner boundary of the myelin sheath using the automatic threshold tool. For each RLN, the total number of fibers, the mean size of fibers and the myelin thickness were determinated.

#### GFP labeling of transplanted cells

This evaluation was performed in 2 others rats for each treated group (reinervated-OB treated and reinnervated-OM treated groups), to determine if the cells injected act in penetrating into the RLN.

Briefly, five days before transplantation, OB and OM cultures were infected with a lentiviral vector harboring enhanced GFP (multiplicity of infection: 20, time of exposure overnight). Also, GFP positive cells were injected as previously described in “materials and methods”.

Three weeks after transplantation of GFP positive cells, rats were euthanized (overdose of pentobarbital), RLN were removed and cryo-conserved (frozen with liquid nitrogen and stored at −80°c). Then, fifteen micrometers sections were coverslipped with mounting media (VectaShield, Vector Laboratories, Burlingame, CA.) and were examined under fluorescent microscope (×100) (Zeiss, Axioscope). Twenty consecutive longitudinal sections were examined for each RLN. For interpretation, fluorescence was compared to control RLN (left RLN no transplanted with GFP positive cells).

### Statistics

Prism® software (Graphpad Software, La Jolla, CA.) was used for statistics. All data were presented as means ± SEM. One way ANOVA was used, followed by a Tukey's test, to identify differences between the groups. A *p* value of <0.05 was considered statistically significant.

## Results

Two months after surgery, videolaryngoscopy, EMG recordings and morphometric analysis were performed blindly in ten rats for each group.

### General observations

In agreement with in our previous observations [9], two months after surgery, animals that had received transplantation of OM cultures (OM and OM+OB treated groups) presented a very slight fibrosis in comparison to the other groups with or without cellular transplantation (not shown).

### Videolaryngoscopy

Two months after surgery, angular measure, amplitude and functionality of movements were assessed.

**Figure 1:**
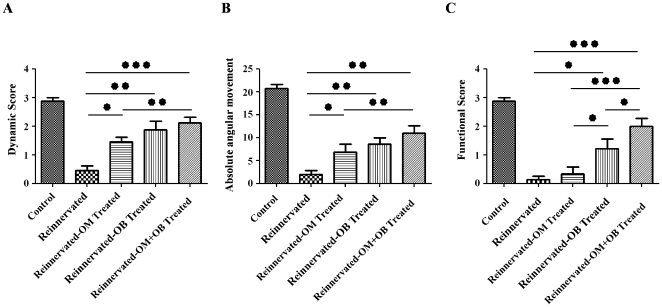
Analysis of dynamic, angular movement and functional recovery. Two months after surgery, videolaryngoscopy analysis permitted to observe an increase in dynamic movement (**A**) and angular measure (**B**) in presence of cellular transplantation (OM, OB and OM+OB cultures) in comparison to reinnervated control group. This analysis permitted also to observe an increase in functional recovery (**C**) in presence of OB and OM+OB cultures in comparison to reinnervated control and reinnervated-OM treated groups, and that co-transplanted OECs (OM+OB) permit to enhance, significantly, functional recovery in comparison to OB-treated group. Data are presented as mean±SEM. Statistical evaluations were based on one way ANOVA test, (*P<0.05, **0.01<P<0.001, ***P<0.001).

Angular measurement and dynamic movement score showed that the reinnervated control group (1.90±1.38°) displayed a significant reduction of movement in comparison with the control group (20.66±1.67°) (p<0.001) (Fig. 1A and 1B). Transplantation of OECs from the mucosa or from the olfactory bulb improves significantly movements of the vocal cords (Fig. 1A and 1B). The reinnervated-OM treated group showed a significant augmentation of the angular movement (6.83±3.96°) (p<0.05) and a significant improvement of the dynamic score in comparison to the reinnervated control group (p<0.05) (Fig. 1A and 1B). Transplantation of OECs from OB increased significantly the angular measure (8.57±3.46°) (0.01<p<0.001) and the dynamic score in comparison to the reinnervated control group (0.01<p<0.001) (Fig. 1A and 1B). Analysis of angular measure and dynamic movement evidenced a great potential recovery of co-transplantation of OECs from OM and OB cultures. Indeed, the reinnervated-OM+OB treated group showed a significant augmentation of the angular movement of the vocal cords (10.95±3.50°) (0.01<p<0.001) and a very significant improvement of the dynamic score (p<0.001) in comparison to the reinnervated control group. Moreover, co-transplantation of OECs from OM+OB improved significantly the dynamic score and the angular movement in comparison to the reinnervated-OM treated group (0.01<p<0.001).

To complete these measurements, using a scale based on the functional recovery, parodoxical movements were analyzed. This analysis revealed that transplantation of OECs from OB improved functional recovery in comparison to the reinnervated control and reinnervated-OM treated (p<0.05) groups and that animals which received OECs obtained from OM did not show any functional recovery in comparison with reinnervated control group (Fig. 1C). This analysis showed also, that co-transplantation of OECs from OM+OB improved very significantly functional recovery in comparison to the reinnervated control (p<0.001) and reinnervated-OM treated groups (p<0.001). Furthermore, the reinnervated-OM+OB treated group presented a significant improvement of functional recovery in comparison to the reinnervated-OB treated group (p<0.05).

For all measurements, control (sham) group presented significantly better movements (angular movements, dynamic and functional scores) in comparison with all the reinnervated groups (p<0.001).

### Electromyography

**Figure 2:**
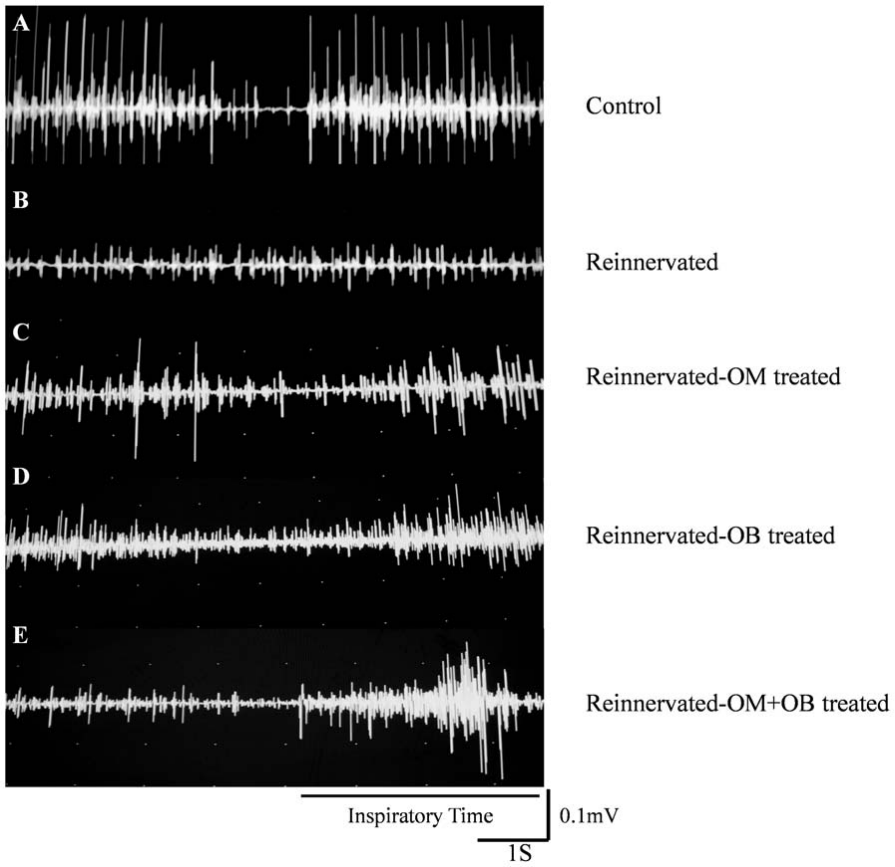
Examples of EMG traces obtained during inspiratory effort in posterior cricoarytenoid muscle. Animals of the sham group presented a typical rich electrical muscular activity, with an increase during inspiration (**A**). EMG traces showed that cellular transplantation of OECs from OM (**C**), OB (**D**) and OM+OB (**E**) improved electrical activities of the laryngeal muscles in comparison to the reinnervated control group (**B**). In particular, the traces presented an augmentation of the electrical muscular activity during inspiration phases whereas reinnervated control animals left this synchronized activities.

Animals of the sham group presented a typically rich electrical muscular activity. This muscular activity was improved during inspiration showing a synchronization of the laryngeal muscles with respiratory cycles (Fig. 2A).

Analysis of the EMG traces showed that cellular transplantation of OECs (from OM, OB and OM+OB) improved electrical activities of the laryngeal muscles in comparison with the reinnervated control group (Fig. 2C, 2D and 2E), in particular the traces presented an augmentation of the electrical muscular activity during inspiration phases whereas reinnervated control animals left this synchronized activities (Fig. 2B).

Typical traces of the different groups are presented in Fig. 2.

**Figure 3:**
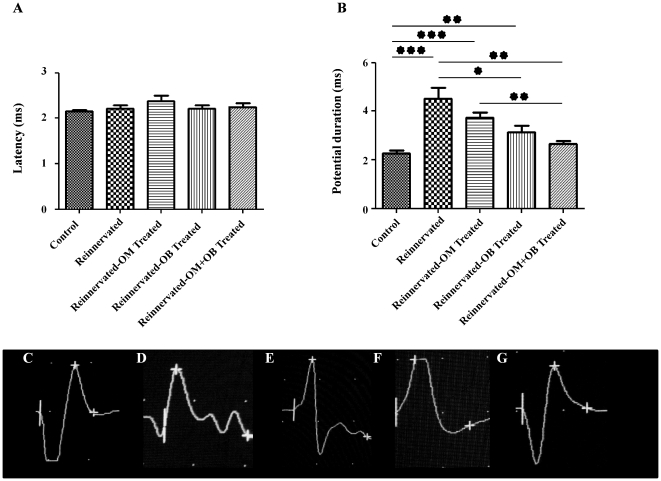
Measure of latency and potential duration. Two months after surgery, analysis of the latency after stimulation showed no difference between the treated groups and the control group (**A**). Potential duration was also measured and showed that reinnervated control group displayed a significant longer potential duration in comparison to sham group (**B**). This analysis permitted, also, to determinate that treated groups showed a reduction in potential duration in comparison to the reinnervated control group (**B**), in particular reinnervated-OB and reinnervated-OM+OB treated groups showed a significant reduction of potential duration with a monophasic response after stimulation (**F and G**). See figure 1 for statistical analysis. Typical observations was presented for the control (**C**), reinnervated control (**d**), reinnervated-OM (**E**), reinnervated-OB (**F**), reinnervated-OM+OB treated (**G**) groups.

Analysis of the EMG traces was completed by measure of latency (Fig. 3A) and potential duration (Fig. 3B). Measure of latency permits to determinate the time that nerve takes to conduct electrical stimulation and measure of potential duration gives information about homogeneity of the nervous conduction in determinating the time during which laryngeal muscles received the electrical stimulation.

Analysis of latencies, in this model of section/anastomosis of the RLN, showed that there is no difference between the different groups, probably due to the small distance between site of denervation and muscular effectors (Fig. 3A). In the same time, potential duration was significantly increased in reinnervated control group (4.53±1.02 ms), with a polyphasic trace (Fig. 3D), in comparison to the control group (2.28±0.27 ms) (p<0.001).Transplantation of OECs from OM (3.74±0.37 ms), OB (3.15±0.46 ms) and OM+OB (2.65±0.31 ms) permitted to reduce potential duration in comparison to the reinnervated control group (4.53±1.02 ms), with a significant reduction for transplanted groups with OECs from OB (p<0.05) and co-transplanted with OECs from OM+OB (0.01<p<0.001) (Fig. 3B). Reinnervated-OM treated group showed a reduction of potential duration in comparison to the reinnervated control group (p = 0.13) with no significant differences, but traces presented also a polyphasic response (Fig. 3E). In contrast, reinnervated-OB treated group showed a reduction of potential duration in comparison to reinnervated-OM treated group (p = 0.13) with no significant differences and in this group, responses presented monophasic profile (Fig. 3F), as observed in control group (Fig. 3C). Reinnervated-OM+OB treated group showed a significant reduction of potential duration in comparison to the reinnervated-OM treated group (0.01<p<0.001), with typical monophasic trace (Fig. 3G) and showed a reduction of potential duration with no significant differences (p = 0.10) in comparison with reinnervated-OB treated group.

### Morphometric analysis for axonal counting

**Figure 4:**
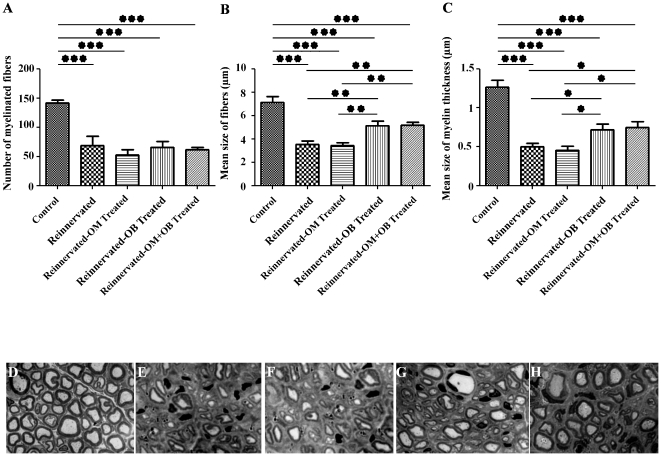
Histological nervous studies. Analysis of the numbers of the myelinated fibers showed that the control group presented a higher number of myelinated fibers in comparison to the treated groups, and that there is no difference between these groups (**A**). Analysis of the mean size of the fibers, two months after surgery, permitted to determinate, that the transplantation of OB and OM+OB cultures increased significantly the mean size of the fibers in comparison to the reinnervated control and reinnervated-OM treated groups (**B**). Analysis of the mean size of the myelin thickness permitted to show that the augmentation of the mean size of the fibers in reinnervated-OB and reinnervated-OM+OB was correlated to an augmentation of the myelin thickness in these groups in comparison to the reinnervated control and reinnervated-OM treated groups (**C**). Typical observations was presented for the control (**D**), reinnervated control (**E**), reinnervated-OM (**F**), reinnervated-OB (**G**), reinnervated-OM+OB treated (**H**) groups (Magnification 630×). See figure 1 for statistical analysis.

The morphological analysis was performed two months after surgery on the distal part of the RLN. At this time, the total number of fibers, the mean size of fibers and the myelin thickness were determinated. The analysis of the numbers of myelinated fibers showed that there is no difference between all the treated groups and that all these groups presented a significant reduction of the numbers of fibers in comparison to the control group (p<0.001) (Fig. 4A).

Interestingly, analysis of the mean size of the fibers and the mean size of the myelin thickness showed that transplantation of OECs from OB and co-transplantation of OECs from OM+OB improved significantly these parameters. In fact, the reinnervated-OB treated group (5.11±1.34 µm) showed a significant augmentation of the mean size of the fibers in comparison to the reinnervated control group (3.54±0.82 µm) (0.01<p<0.001) and in comparison with the reinnervated-OM treated group (3.45±0.81 µm) (0.01<p<0.001) (Fig. 4B). Reinnervated-OB treated group (0.72±0.18 µm) showed also an augmentation of the mean size of the myelin thickness in comparison to the reinnervated control (0.49±0.15 µm) (p<0.05) and reinnervated-OM treated group (0.46±0.23 µm) (p<0.05) (Fig. 4C). Co-transplantation of OECs from OM and OB permitted also to significantly improve the mean size of fibers (5.21±0.75 µm) in comparison to reinnervated control and reinnervated-OM treated groups (0.01<p<0.001) (Fig.4B), and the mean size of the myelin thickness (0.75±0.25 µm) in comparison to these groups (p<0.05) (Fig. 4C).

In summary, transplantation of OECs from OB resulted in an enhancement of size of the fibers and the thickness of the myelin in comparison to the reinnervated control and reinnervated-OM treated groups, with no additive effects in case of co-transplantation of OB+OM OECs. Thus, transplantation of OB-OECs alone appears responsible for the observed beneficial effects on axonal regrowth.

Typical observations were presented for the control (Fig. 4D), reinnervated control (Fig. 4E), reinnervated-OM (Fig. 4F), reinnervated-OB (Fig. 4G) and reinnervated-OM+OB (Fig. 4H) treated groups.

### GFP labeling of transplanted cells

Three weeks after OB-GFP cells (n = 2) and OM-GFP cells (n = 2) transplantation, the right RLN was removed and twenty consecutive sections of fifteen micrometers were performed and examined under fluorescent microscope. It appeared, as previously described by our laboratory [9], [14], that any GFP positive cells could be observed into the nerve which received cellular transplantation. For observation, RLN control was used. Typical observations were presented for RLN transplanted with GFP positive cells (injection around the nerve, in matrigel) (Figure S2A) and compared with sciatic nerve transplanted (intra-nervous injection) with GFP positive cells (Figure S2B; taken from material published in [14]).

## Discussion

To our knowledge, this is the first reported study which presents co-transplantation of the two sub-populations of OECs after nerve lesion. Also, this study confirms our previous results regarding the different abilities that could be played by OECs originating from OB and OM [8], [9].

The co-transplantation model presented here demonstrates the complementary effects played by OM-OECs and OB-OECs. These two populations of glial cells injected separately improve vocal cord movements and electrical activity of the laryngeal muscles, with an effect on fibrosis associated with aberrant movements in the case of OM-OECs transplantation and an improvement on axonal regrowth associated with a partial functional recovery in the case of OB-OECs transplantation. At the same time, co-transplantation of OM-OECs and OB-OECs permits, not only to conserve their specific characteristics, but also permits to enhance abilities of each sub population. Indeed, the co-transplanted group presents an impressive functional recovery of the vocal cords movements.

The choice was made to inject OECs with their specific microenvironment due to the main role of this described elsewhere. Indeed, others researchers report the presence of olfactory nerve fibroblasts, which them OECs are closely associated *in vivo*, as casting for effective transplants [15], [16], [17]. Nevertheless, it could be excluded that a part of the effects described here may be due to contaminating cells. Differences in culture conditions might explain some contradictory results, even by our team, that could be observed after OECs transplantation [3], [18].

In this study, we chose a laryngeal model for the evaluation because this model permits to score the functionality of the regeneration, based on the observation of aberrant movements and synkinesis. In fact, section/anastomosis of the recurrent nerve leads to misdirected reinnervations of antagonist muscles and muscular co-contraction phenomena [12]. Moreover, lesion of the recurrent nerve was specifically chosen because this model permits to perform a selective motor denervation of the laryngeal muscles, RLN is a mixed nerve. It also permits evaluation at an early stage (two months) in comparison to the vagus nerve lesion model (three months), due to the short distance between the denervation site and muscular targets (7 mm). In our study, an injection around the sutured nerve was chosen because the size of the RLN does not permit an intra-nervous injection of the cellular solution.

Finally, it appears that, as previously described, no GFP positive cell could be found into the anastomosed RLN, indicating that OM-OECs and OB-OECs exert their specific effect in interacting with microenvironment but not in penetrating into the severed nerve [9], [14]. In fact, in our model, RLN size did not permit an injection into the nerve, that's why cellular cultures were laid over the section/anastomosis site.

Therapies based on the use of OECs have been widely studied in many nerve lesion situations [3], [5], [19], [20], [21], in particular to treat spinal cord or peripheral nerve injuries. Recently, it was discovered that OECs are neural crest cells and share a common developmental heritage with Schwann cells. This could explain the great ability of these cells to enhance recovery after transplantation in PNS [22], [23], [24]. However, knowledge about these unique glial cells remains imperfect and it is important to well define properties of these prior to clinical use. For this purpose, the use of OECs obtained from OM seems to be the only acceptable source. However, despite the fact that, OB-OECs and OM-OECs express the same phenotypic markers *in vitro* [25], it seems that they do not have the same ability to migrate and interact with microenvironment after transplantation *in vivo* [7]. Our recent studies have clearly demonstrated that these two sub-populations of OECs are specifically involved in differential physiological processes both *in vitro* [8] and *in vivo* [9].

OM-OECs express typical biological properties. In particular, OM-OECs could improve recovery by regulation of inflammatory responses and extracellular matrix formation as well as an increase in angiogenesis [26]. However, as previously described, by other reported studies, it seems that these cells have no specific ability to improve axonal regrowth [27], [28], [29]. In contrast, OB-OECs exert their specific abilities to improve functional recovery by inducing targeted axonal regrowth. These distinct behaviors of OM-OECs and OB-OECs could be due to their role during neurogenesis of the primary olfactory neurons. Indeed, as well described by Windus et al., OECs display typical organization in lamina propria and in OB, in particular in their opposite relationship with axons of primary olfactory neurons [6]. OM-OECs seem to enhance growth of olfactory neurons through lamina propria, whereas OB-OECs seem, for a part of them, participate to axonal targeting into olfactory glomeruli [6].

In our study, it appears that these distinct properties could be complementary to improve functional recovery and axonal regrowth in cases of peripheral nerve injury. In this co-transplantation model, each sub-population of OECs preserves their specificity and could, by an additive effect, enhance functional recovery. Indeed, co-transplanted animals presented a slight fibrosis, typical of the main role played by OM-OECs, and in the same time, these animals showed an increase of the mean size of the fibers and the myelin thickness, in agreement with the observations of the OB-treated animals. These distinct abilities permit to the transplanted animals to present the best results in all the parameters studied, in particular about functional recovery.

This work underlines the fact that for complete functional recovery after nerve lesions, further investigations could be conducted to optimize the abilities of olfactory ensheathing cells.

## Supporting Information

Figure S1 **These images represent typical endoscopic views of the rat glottis plan.** During evaluations, landmarks are determined to have the same view for each recording. To this view we could measure the maximal abduction (**A**) and the maximal adduction (**B**).
(TIF)

Figure S2 **Recurrent laryngeal nerve transplanted with GFP labeled cells (green fluorescence) (A) as compared with GFP labeled OB-OECs retained at the lesion site into crushed rat's sciatic nerve (B; taken from material published** [**14**] **).** Magnification ×100.
(TIF)
