# Supplementary figures and images for: Co-Transplantation of Olfactory Ensheathing Cells from Mucosa and Bulb Origin Enhances Functional Recovery after Peripheral Nerve Lesion
Source: PLoS One. 2011 Aug 3;6(8):e22816. doi: 10.1371/journal.pone.0022816 (PMC3149611; doi:10.1371/journal.pone.0022816)

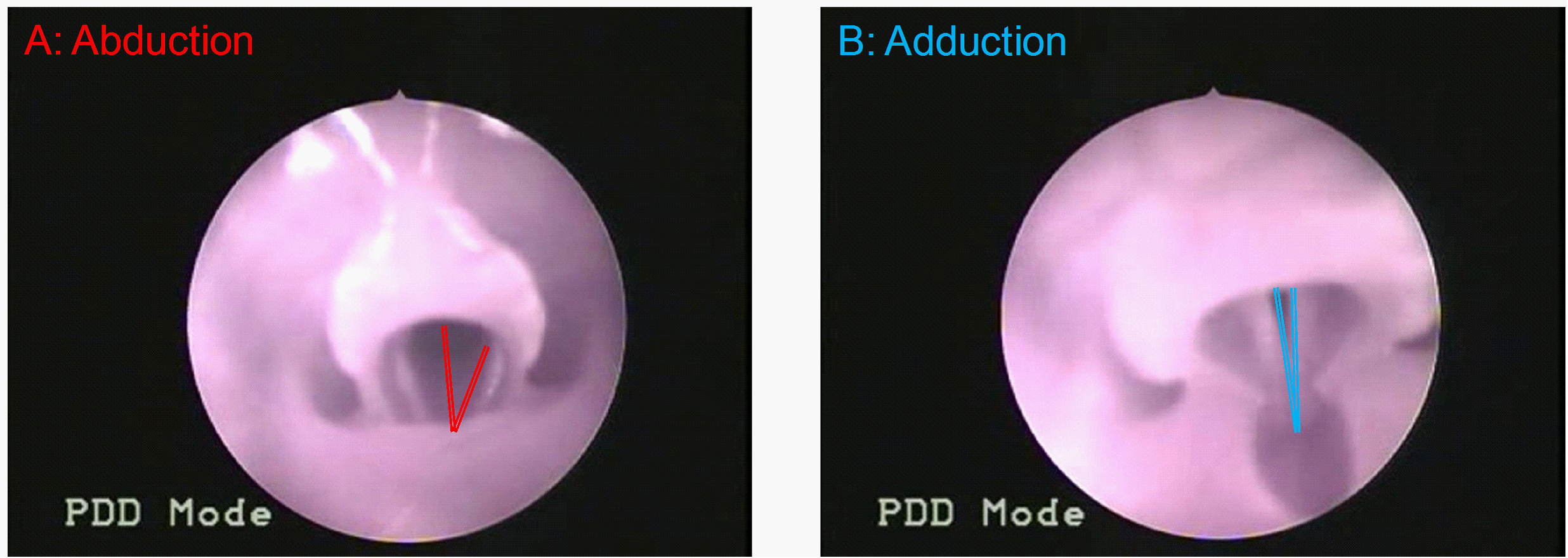

Supplement: Figure S1 — These images represent typical endoscopic views of the rat glottis plan. During evaluations, landmarks are determined to have the same view for each recording. To this view we could measure the maximal abduction (A) and the maximal adduction (B). (TIF) [file pone.0022816.s001.tif]

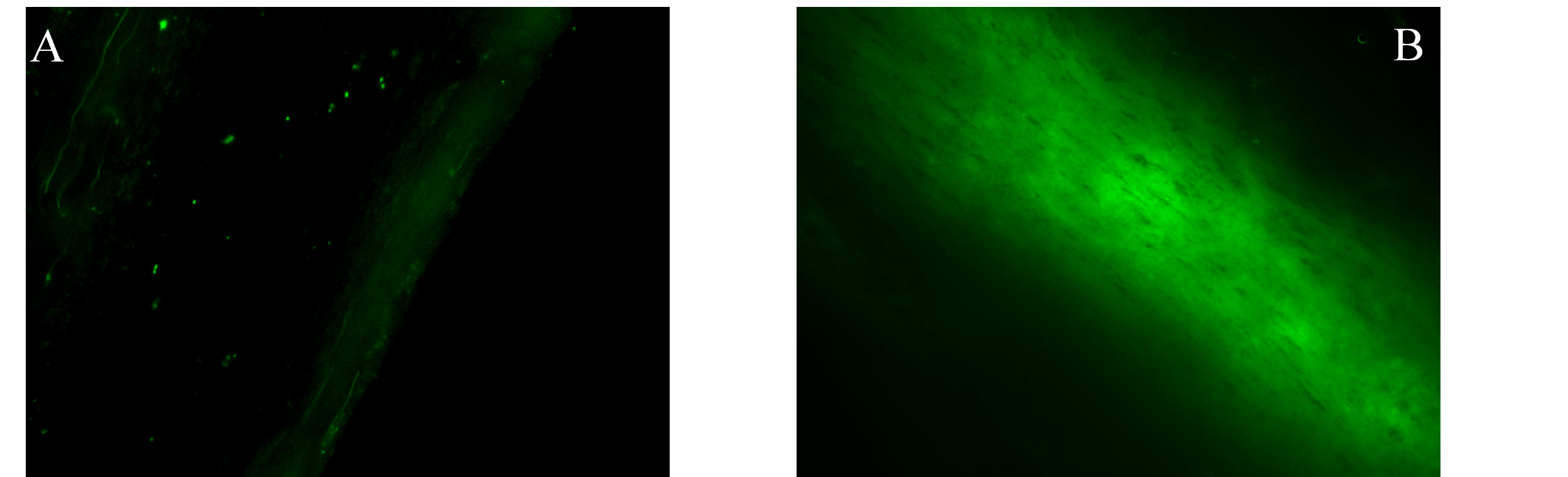

Supplement: Figure S2 — Recurrent laryngeal nerve transplanted with GFP labeled cells (green fluorescence) (A) as compared with GFP labeled OB-OECs retained at the lesion site into crushed rat's sciatic nerve (B; taken from material published [14] ). Magnification ×100. (TIF) [file pone.0022816.s002.tif]
